# Zuotai (β-HgS)-containing 70 Wei Zhen-Zhu-Wan differs from mercury chloride and methylmercury on hepatic cytochrome P450 in mice

**DOI:** 10.12688/f1000research.40667.2

**Published:** 2021-06-24

**Authors:** Yu Nie, Shang-Fu Xu, Yan-Liu Lu, Xiu-Rong Zhao, Cen Li, Li-Xin Wei, Jie Liu

**Affiliations:** 1 Key Laboratory for Basic Pharmacology of Ministry of Education and the Joint International Research Laboratory of Ethnomedicine of Ministry of Education, Zunyi Medical University, Zunyi, 563006, China; 2 Research Lab, Zunyi Blood Center, Zunyi, 563000, China; 3 Key Lab of Tibetan Medicine, Northwest Institute of Plateau Biology, Chinese Academy of Sciences, Xining, Qinghai, 810001, China

**Keywords:** Zuotai, 70 Wei-Zhen-Zhu-Wan (Rannasangpei, Qishiwei), HgCl2, MeHg, Cytochrome P450, Nuclear receptors.

## Abstract

**Background: **Zuotai (mainly β-HgS)-containing 70 Wei-Zhen-Zhu-Wan (70W, *Rannasangpei*) is a famous Tibetan medicine for treating cardiovascular and gastrointestinal diseases.  We have shown that 70W protected against CCl _4_ hepatotoxicity.  CCl _4_ is metabolized via cytochrome P450 (CYP) to produce reactive metabolites. Whether 70W has any effect on CYPs is unknown and such effects should be compared with mercury compounds for safety evaluation.

**Methods: **Mice were given clinical doses of 70W (0.15-1.5 g/kg, po), Zuotai (30 mg/kg, po), and compared to HgCl _2 _(33.6 mg/kg, po) and MeHg (3.1 mg/kg, po) for seven days. Liver RNA and protein were isolated for qPCR and Western-blot analysis.

**Results: **70W and Zuotai had no effects on hepatic mRNA expression of Cyp1a2, Cyp2b10, Cyp3a11, Cyp4a10 and Cyp7a1, and corresponding nuclear receptors [aryl hydrocarbon receptor (AhR), constitutive androstane receptor (CAR), pregnane X receptor (PXR), peroxisome proliferator-activated receptor-α (PPARα); farnesoid X receptor (FXR)]. In comparison, HgCl _2 _and MeHg increased mRNA expression of Cyp1a2, Cyp2b10, Cyp4a10 and Cyp7a1 except for Cyp3a11, and corresponding nuclear receptors except for PXR. Western-blot confirmed mRNA results, showing increases in CYP1A2, CYP2B1, CYP2E1, CYP4A and CYP7A1 by HgCl _2 _and MeHg only, and all treatments had no effects on CYP3A.

**Conclusions: **Zuotai and Zuotai-containing 70W at clinical doses had minimal influence on hepatic CYPs and corresponding nuclear receptors, while HgCl _2 _and MeHg produced significant effects.  Thus, the use of total Hg content to evaluate the safety of HgS-containing 70W is inappropriate.

## Introduction

Tibetan Medicine is one of the important medical heritages of the world ^1^. Zuotai, a Tibetan medicine mixture containing β-HgS, has been included in many famous Tibetan medicines for the treatment of diseases ^2– 4^. A systematic review of available studies of Tibetan medicine, however, indicates that the literature in Western industrialized countries is scarce ^5^. Traditional Tibetan medicines use polyherbo-metallic mixture recipes as opposed to a single ingredient in the treatment of diseases. For example, in a review of 193 herbo-metallic Tibetan medicine recipes for liver diseases, herbs/plants (181 kinds), animal products (7 kinds), and minerals (5 kinds) were frequently used ^6^. Well-designed pharmacology and clinical studies are encouraged to elucidate the pharmacology, safety, and clinical efficacy of Tibetan medicines ^5, 6^.

We have recently indicated that chemical compositions of minerals (metals) are a major determinant of their therapeutic effects and toxicity in Tibetan medicines ^7^. 70W Zhen-Zhu Wan (70W, also called *Rannasangpei,* Qishiwei) is such an example 8. 70W was developed in the middle of fifteenth century and is composed of herbo-metallic mixtures, mainly from pearl, Hong-sik, *Albergia odorifera*, Nine stone, Saffron, Bezoar, Musk and Zuotai (a mineral mixture) in the treatment of cardiovascular, gastrointestinal, and neurodegenerative diseases ^8^, and is listed in the 2015 edition of Pharmacopoeia of China ^9^. 70W is effective experimentally against vascular dementia in rats ^10^, and protects cerebral ischemia-reperfusion injury via blood-brain barrier and metabonomics with 18 identified active ingredients ^11^. We have recently demonstrated that 70W is effective in protecting against LPS plus MPTP-induced chronic neuroinflammation and dopaminergic neuron loss ^8^ and could modulate gut microbiota as a means of protection ^8, 12^. 70W dose-dependently protected against CCl _4_-induced liver injury, probably by activation of the Nrf2 antioxidant pathway ^13^.

CCl _4_ is metabolized via cytochrome P450 (CYP450), particularly CYP2E1, to produce reactive metabolites ^14^. Whether the protective effects of 70W against CCl _4_ hepatotoxicity is related to CYP450 inhibition is not known. In addition, 70W might be used in combination with other medications since it has many beneficial effects because it contains many ingredients. It has the potential to cause herb-drug interactions, especially on the liver CYP450 gene, similar to other Chinese medicine formulae ^15^. CYPs are the mixed function oxidase system mainly existing in the liver, and play roles in the metabolism of over 80% drugs ^16^. Induction or inhibition of CYP450 is implicated in traditional medicine-induced hepatoprotection and/or hepatotoxicity ^15, 17, 18^. CYP450 genes are regulated by corresponding nuclear receptors, their coordinated regulation affects hepatic phase I and phase II metabolisms ^19^. 

This study was therefore designed using 1–5 times clinical doses of 70W (0.15, 0.5 and 1.5 g/kg, po) for oral administration to mice for 7 days and comparing its effects with equivalent Hg contents of Zuotai, HgCl _2_, and 1/10 Hg contents of MeHg, in an attempt to obtain information for the safe use of Zuotai-containing 70W in the clinic.

## Methods

### Reagents

70W and Zuotai was provided by Tibetan Medicine Manufacture Factory as described previously ^8^, based on the 2015 edition of Pharmacopoeia of China for QA/QC control (Lot number Z20110561). 70W was prepared by grinding the pill into powder, adding distilled water to prepare the suspension for oral administration. Mercury chloride (HgCl _2_ Cat# M1136) and methylmercury (MeHgCl Cat# 442534) were from Sigma (St. Louis, MO, USA). All other chemicals were commercially available reagents.

### Animals

Male Kunming mice (20 ± 2 g) were purchased from Animal Experimental Center of theThird Military Medical University (Chongqing, China). Animals were maintained in the SPF-grade facilities at Zunyi Medical University, with a controlled environment (22 ± 1°C, 50 ± 2% humidity and a 12 h: 12 h light: dark cycle) and free access to purified water and standard laboratory feed. Efforts were made to ameliorate distress and harm to animals by daily monitoring and humane treatment of the animals. To reduce the use of animals, the minimal number of mice (n=5)/group according to the experiment requirement was used which are sufficient for statistical analysis. All animal care and experimental protocols are complied with the Animal Management Guidelines of the Chinese Ministry of Health and approved by Animal Use and Care Committee of Zunyi Medical University (2015-07).

### Animal treatments

Mice were randomly divided into seven groups of five mice each (Total number n=35), respectively as the control, 70W (0.15, 0.5, 1.5g/kg), Zuotai (30 mg/kg, the amount contained in 70W), HgCl _2_ (33.6 mg/kg, equivalent Hg as HgS) and MeHgCl (MeHg, 3.1 mg/kg, 1/10 of Hg). Mice were given oral administration for seven consecutive days. The dose regimen selection was based on our prior publications for 70W (at clinical dose) ^8^ or for zuotai and mercury compounds ^20^. Twenty-four hours after the last dose, the animals were euthanized and the livers were collected and stored at 80°C prior to analysis.

### Liver toxicity evaluation

The activities of alanine aminotransferase (ALT) and aspartate aminotransferase (AST) were determined by commercial kits (Jaingcheng, Nanjing, China) ^21^. Liver samples were fixed in 10% formalin prior to routine processing and paraffin embedding. Liver sections (4 µm) were dewaxed in xylene, rehydrated in different concentrations of alcohol (100%, 95%, 80%, 75%) and stained with hematoxylin, followed by counterstaining with eosin. After rinsing, the slides were rehydrated with series of alcohol (75%, 95%. 100%) and mounted with cover glass slip. The slides were examined in nine random fields under a light microscope (Leica Microsystems Ltd., Wetzlar, Germany) ^21, 22^．

### Real-time PCR

Approximately 50–100 mg of tissue was homogenized in 1 ml TRIzol (TakaRa Biotechnology, Dalian, China) and the total RNA was extracted according to manufacturer’s instructions. The quality and quantity of RNA were determined by the Nanodrop (Thermo Scientific, ND-2000, USA), with 260/280 ratio >1.8. Total RNA was reverse transcribed with a High Capacity Reverse Transcriptase Kit (Applied Biosystems, Foster City, CA, USA). The primers were designed with Primer3 software and listed in Table 1．

**Table 1.** Primer sequences for real-time RT-qPCR.

	Access#	Forward	Reverse
*AhR*	NM_013464	ACCAGAACTGTGAGGGTTGG	CTCCCATCGTATAGGGAGCA
*β-actin*	NM_007393	GATCTGGCACCACACCTTCT	GGGGTGTTGAAGGTCTCAAA
*CAR*	NM_009803	CTCAAGGAAAGCAGGGTCAG	AGTTCCTCGGCCCATATTCT
*Cyp1a2*	NM_009993	AATGTCACCTCAGGGAATGC	GCTCCTGGACAGTTTTCTGC
*Cyp2b10*	NM_009999	AAGGAGAAGTCCAACCAGCA	CTCTGCAACATGGGGGTACT
*Cyp2e1*	NM_021282	GGACGCTGTAGTGCATGAGA	CAACTGTACCCTTGGGGATG
*Cyp3a11*	NM_007818	AGGGAAGCATTGAGGAGGAT	GGTAGAGGAGCACCAAGCTG
*Cyp4a10*	NM_010011	CACACCCTGATCACCAACAG	TCCTTGATGCACATTGTGGT
*Cyp7a1*	NM_007824	CAACGGGTTGATTCCATACC	ATTTCCCCATCAGTTTGCAG
*FXR*	NM_001163700	TGGGTACCAGGGAGAGACTG	GTGAGCGCGTTGTAGTGGTA
*PPARa*	NM_011144	GTCCTCAGTGCTTCCAGAGG	GGTCACCTACGAGTGGCATT
*PXR*	NM_010936	CCCATCAACGTAGAGGAGGA	TCTGAAAAACCCCTTGCATC

The 15 µL PCR reaction mix contained 3 µL of cDNA (10 ng/µL), 7.5 µL of iQ ^TM^ SYBR Green Supermix (Bio-Rad Laboratories, Hercules, CA), 0.5 µL of primer mix (10 µM each), and 4 µL of ddH _2_O. After 5 min denature at 95°C, 40 cycles were performed: annealing and extension at 60°C for 45 seconds and denature at 95°C for 10 seconds. Dissociation curve was performed after finishing 40 cycles to verify the quality of primers and amplification. Relative expression of genes was calculated by the 2 ^-ΔΔCt^ method and normalized to the house keeping gene β-actin or expressed as a percentage of controls ^8, 21^.

### Western blot analysis

Approximately 80 mg of liver tissue was homogenized with RIPA lysis buffer containing 1 mM PMSF and freshly prepared proteinase inhibitors. The homogenates were centrifuged at 12,000 g at 4⍛C for 10 min, and the protein concentration in the supernatants was determined by the BCA assay, and denatures at 90⍛C for 10 min with Nupage loading buffer. Approximately 30 µg proteins were separated in the 10% Nupage gel and transferred to the PVDF membrane. The membranes were blocked in 5% of the skim milk for 1 hour at room temperature, followed by incubation with primary antibodies (CYP1A2 (1:500), CYP2B1 (1:500), CYP2E1 (1:500), CYP3A4 (1:500), CYP4 (1:500), CYP7A1 (1:500), and GAPDH (1:2000)) at 4°C overnight. After washing the membranes with TBST four times, the secondary horseradish peroxidase (HRP) labelled anti-rabbit, or anti-mouse antibodies were added (1:5000) (Beyotime, Shanghai, China), and incubated at room temperature for 1 hour. The enhanced chemiluminescent reagents (ECL) were used to detect the intensity of protein-antibody complexes, and intensity was semi-quantified with Quantity One software (Bio-Rad, USA) ^18^.

### Statistical analysis

Data were expressed as mean and standard error. SPSS 19 was used for statistical analysis. Data were analyzed using a one-way analysis of variance (ANOVA), followed by Duncan’s multiple range test, and a *p* value < 0.05 was considered significant.

## Results

### Animal general conditions

At the doses of 70W and Zuotai used in the present study, animals were healthy, without body weight loss and no mortality occurred. No significant elevations of serum ALT and AST were evident, and histology did not reveal overt lesions ^12, 20– 22^. HgCl _2_ and MeHg groups showed body weight loss and mild histology lesions, consistent with prior publications ^12, 21, 22^.

### mRNA expression of nuclear receptors and cytochrome P450 genes

**Figure 1.**
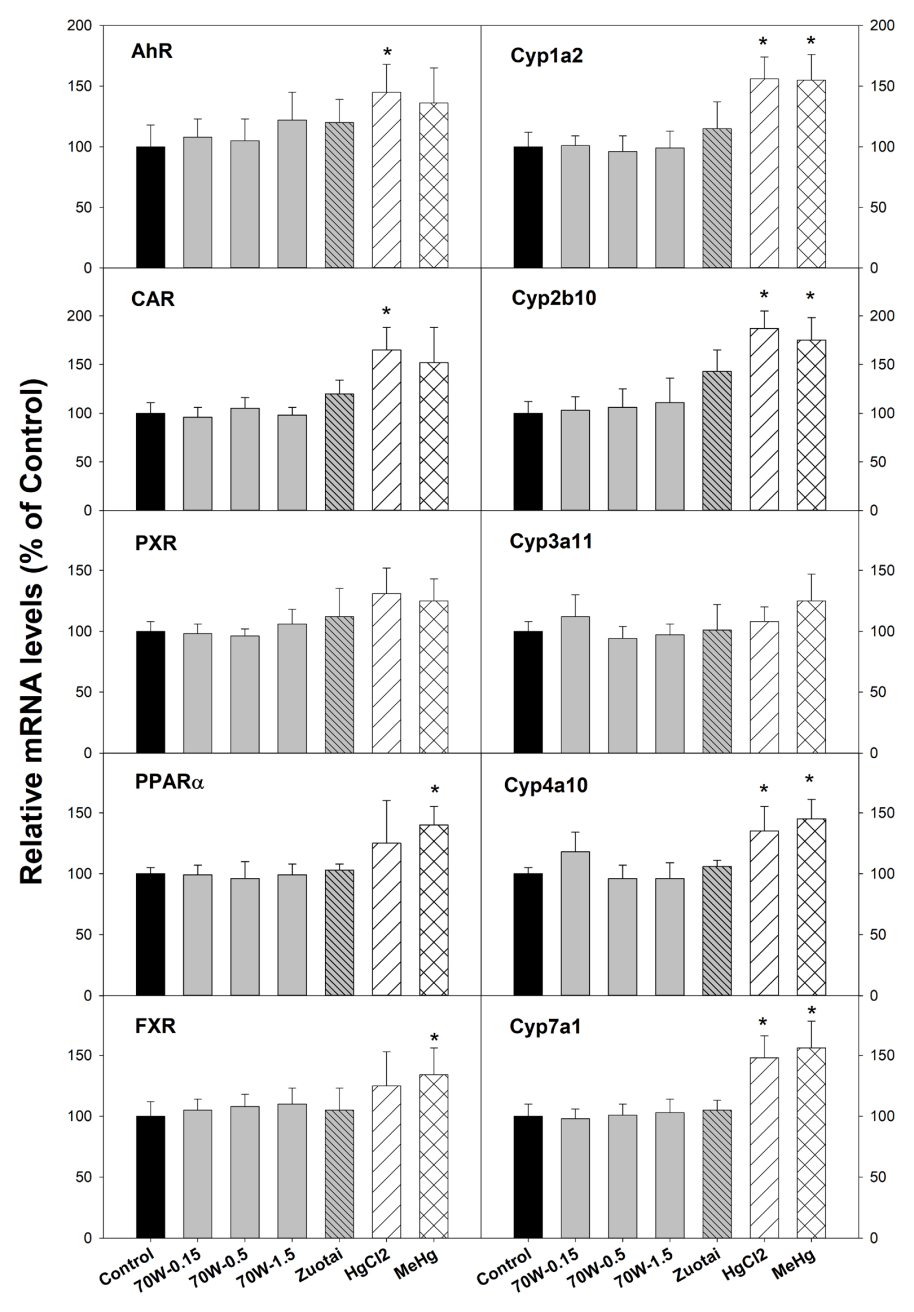
Effect of 70W and mercury compounds on nuclear receptor and corresponding CYP gene expression. Mice were given 70W 0.15, 0.5, and 1.5 g/kg, po. Zuotai (30 mg/kg, po), HgCl _2_ (33.6 mg/kg, po), and MeHg (3.1 mg/kg, po) daily for seven days, and hepatic total RNA and protein were extracted for RT-PCR analysis. Data are mean ± SE, n = 5. *Significantly different from control, *p*< 0.05.

Figure 1 illustrates mRNA expression of nuclear receptors (left side) and cytochrome P450 isozyme genes (right side). The aryl hydrocarbon receptor (AhR) mainly mediates the expression of CYP1A enzymes such Cyp1a2; Constitutive androstane receptor (CAR) mediates CYP2 enzymes such as Cyp2b10; Pregnane X receptor (PXR) plays an important role in the regulation of CYP3A enzymes such as Cyp3a11; Peroxisome proliferator-activated receptor α (PPARα) regulates induction of CYP4A enzymes such as Cyp4a10 ^19^. The Farnesoid X receptor (FXR) regulates cholesterol 7α hydroxylase (Cyp7a1) induction ^23^. The results show that compared with the controls, 70W at 0.15, 0.5, and 1.5 g/kg doses and Zuotai (β-HgS, 30 mg/kg) had no effects on these nuclear receptor and CYP gene expressions. In contrast, HgCl _2_ (33.6 mg/kg) and MeHg (3.1 mg/kg) significantly increased AhR, Cyp1a2, CAR, Cyp2b10, PPARα, Cyp4a10, FXR, and Cyp7a1, while having no significant effects on PXR, Cyp3a11 ( Figure 1).

### Protein expression of cytochrome P450 isozymes

**Figure 2.**
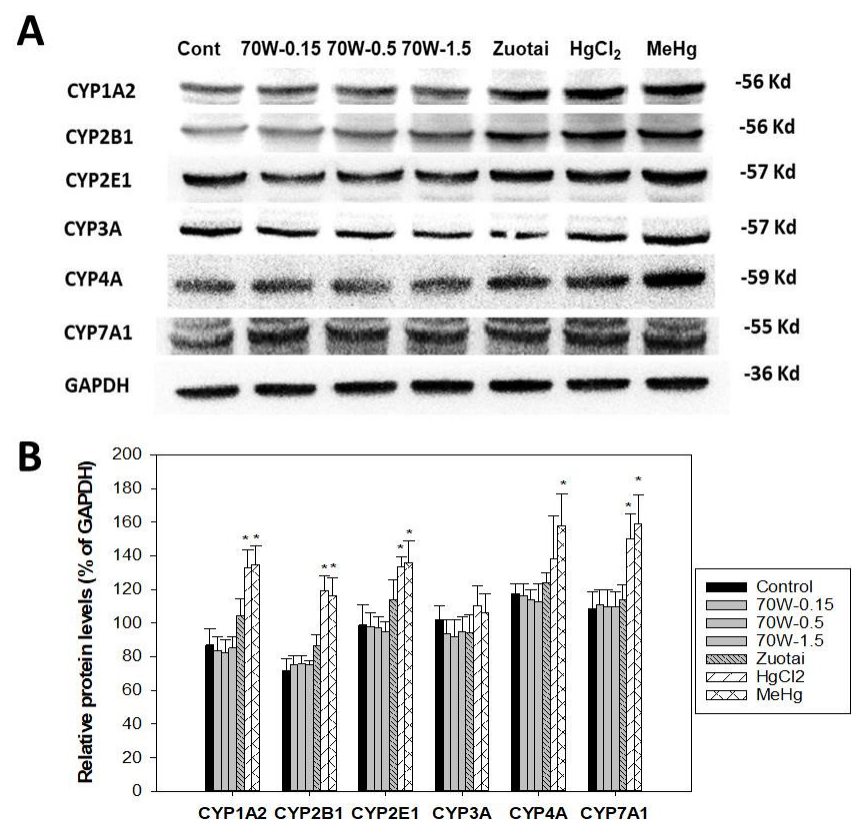
Effect of 70W and mercury compounds on cytochrome P450 isoenzyme protein expression. Mice were given 70W 0.15, 0.5, and 1.5 g/kg, po. Zuotai (30 mg/kg, po), HgCl _2_ (33.6 mg/kg, po), and MeHg (3.1 mg/kg, po) daily for seven days, and hepatic proteins were extracted and pooled for Western-blot analysis. **A**, the representative western-blot; **B**, statistical analysis of P450 proteins. Data are mean ± SE of 3-5 replicates. *Significantly different from control, *p*< 0.05.

Figure 2 illustrates protein expression of P450 isozymes. Figure 2A shows representative western-blots for CYP1A2, CYP2B1, CYP2E1, CYP3A, CYP4A, and CYP7A1; Figure 2B shows the statistical analysis of 3-5 replicates. Consistent with mRNA expression, 70W at 0.15, 0.5, and 1.5 g/kg doses and Zuotai (β-HgS, 30 mg/kg) had no apparent effects on cytochrome P450 isozyme protein expressions. In contrast, HgCl _2_ (33.6 mg/kg) and MeHg (3.1 mg/kg) significantly increased CYP1A2, CYP2B1, CYP4A, and CYP7A1, while having no effects on CYP3A ( Figure 2).

## Discussion

The potential efficacy and toxicity of minerals (metals) in traditional medicines is currently a matter of debate ^7, 24^. In the present research, we examined the effects of β-HgS-containing Zuotai and Zuotai-containing 70W on hepatic CYP 1-4 and CYP-7 families, and their corresponding nuclear receptors, compared to HgCl _2_ and MeHg at both mRNA and protein levels. Briefly, 70W at 1 to 5-times clinical doses and Zuotai (β-HgS, 30 mg/kg, po) administered for seven days did not produce significant effects on the liver CYP450 gene and protein expressions in mice. HgCl _2_ and MeHg at 1/10 Hg dosing increased the expression of CYP1A, CYP2B, CYP2E1, and CYP7A at the mRNA and/or protein levels. These results further demonstrate that chemical forms of metals are a major determinant of their biological effects and that the use of HgCl _2_ or MeHg for risk assessment on minerals in traditional medicines is inappropriate ^7^.

### 70W and Zuotai in Tibetan Medicines

Tibetan medicine has thousands of years of history and is still used in the world today to treat a variety of diseases, including liver diseases ^1– 4, 6^. Herbal-metallic preparations are believed to assist the delivery of drugs to the target, contribute to therapeutic effects, and reduce toxicity ^7^. 70W is a famous Tibetan medicine listed in the 2015 Edition of Chinese Pharmacopoeia for the treatment of various diseases ^3, 10^. The major ingredients in 70W and the mode of the protection against cerebral ischemia-reperfusion injury has recently been demonstrated ^11^. We have shown that 70W is effective against CCl _4_-induced liver injury, protected LPS plus MPTP-induced neurotoxicity ^8^, and modulated gut microbiota ^8, 12^. The present study further demonstrated that the hepatoprotective effects of 70W is not due to the inhibition of CYP450 to reduce CCl _4_ bioactivation, rather the activation of the Nrf2 antioxidant pathway ^13^.

Zuotai is a mineral mixture, with 54% of β-HgS ^25^, and is included in a small amount to many valuable Tibetan medicines ^1– 3^. Mercury (Hg) is a toxic metal; the safety of Hg-containing traditional medicines is of concern ^24^. The chemical speciation, spatial distribution of mercury from Zuotai are different from that of HgCl _2_^7, 25^, resulting in differential toxicity. A recent human study revealed that Zuotai-containing Tibetan medicines are safe at clinical doses ^26– 28^, including 70W ^29^. Indeed, Zuotai differs from HgCl _2_ and MeHg in producing hepatotoxicity ^21^, nephrotoxicity ^30^, and intestinal toxicity with gut microbiome disruptions ^20^. The present study demonstrated that Zuotai-containing 70W at clinical doses had minimal effects on hepatic CYP450, supporting the notion that Zuotai and 70W at clinical doses are safe ^26– 29^. 

### Effects of mercury compounds on cytochrome P450

Cytochrome P450 1A1 (CYP1A1) is a hepatic and extrahepatic enzyme that is regulated by the AhR signaling pathway and is regarded as carcinogen activation CYP450 family ^31^. CYP-1 family includes CYP1A1, CYP1A2, and CYP1B1，and CYP1A1/CYP1A2 has become a therapeutic tool for the bioactivation of prodrugs, particularly cytotoxic agents. Little is known about effects of 70W on CYP1A family. We have shown previously that oral Zuotai (β-HgS) and cinnabar (α-HgS) had minimal effects of hepatic P4501A family gene expression ^32^. However, in rats, Zuotai at higher doses could decrease CYP1A2 activity ^33^. In comparison, the effects of HgCl _2_ on CYP1A expression were more dramatic. In Zebra fish, a low dose (0.1 LC50) of HgCl _2_ increased CYP1A1, but at higher doses (0.4 and 0.8 LC50), the expression of CYP1A1 was suppressed ^34^. In the mouse heart, kidney and lung, HgCl _2_ (2.5 mg/kg, ip) increased CYP1A1, along with other CYP450 isoforms ^35^. In the present study, HgCl _2_ at 33.6 mg/kg increased CYP1A2 at mRNA and protein levels, largely in agreement with the above literature ^32– 35^ In another study, mice that chronically (6 weeks) received HgCl _2_ (32 mg/kg) and MeHg (2.6 mg/kg), had increased expressions of hepatic Cyp1a1 and Cyp1b1, while cinnabar (HgS, 300 mg/kg) and cinnabar-containing An-Gong-Niu-Huang Wan were ineffective ^36^. Thus, the effects of mercury compounds on CYP1 family are dependent on the mercury forms, the dose, route, and duration of administration.

The CYP-2 family is easily induced by many xenobiotics such as phenobarbital. CAR is shown to play a crucial role in the activation of CYP2B genes by xenobiotics ^19^. The CYP-2 family mainly includes the CYP2B subfamily and CYP2E1. CYP2E1 metabolizes an extensive array of pollutants, drugs, and other small molecules, often resulting in bioactivation to reactive metabolites, which in turn damage mitochondria ^37^. HgCl _2_-induced hepatotoxicity and oxidative stress is partially mediated through its effects on CYP2E1 ^38^. HgCl _2_ (2.5 mg/kg, ip) increased the expression of Cyp2b9 and Cyp2b10 in mice hearts ^39^ and HgCl _2_ (33.6 mg/kg, po) increased Cyp2b10 expression in the livers of mice ^32^. Under the present experimental conditions, Cyp2b10 mRNA and CYP2B protein expression were increased by HgCl _2_ and MeHg only. 

CYP3A is the most abundant subfamily of CYP450, with the highest content in the liver and intestines, and is involved in the metabolism of clinical drugs ^17, 18^. CYP3A can be induced or inhibited by a variety of substances. In the present study conditions, 70W and mercury compounds had minimal effects on Cyp3a11 mRNA and CYP3A protein expression. The length of Hg compound administration could make a difference as compared to the present study.

CYP4A is involved in lipid metabolism and is regulated by PPARα, their dysregulations are implicated in xenobiotics induced adverse effects leading to various human diseases ^19^. Researchers found that HgCl _2_ exposure is associated with increased risk of cardiovascular disease and profound cardiotoxicity, and their results show that mercury treatment caused a significant induction of the cardiac hypertrophy markers, along with CYP4A genes (Cyp4a10, Cyp4a12, Cyp4a14) ^35^. In the present study, 70W and Zuotai at 1–5 times clinical doses do not have appreciable effects on PPARα and Cyp4a10 mRNA expression and CYP4A protein expression, while HgCl _2_ and MeHg increased PPARα and Cyp4a10 mRNA, as well as CYP4A protein, consistent with our prior observation that HgCl _2_ increased PPARα and Cyp4a10 in livers of mice after seven days of administration ^32^. In mice chronically (6 weeks) dosed with HgCl _2_ (32 mg/kg) and MeHg (2.6 mg/kg), the expression of Cyp4a10 was increased, but cinnabar (HgS, 300 mg/kg) and cinnabar-containing An-Gong-Niu-Huang Wan was ineffective ^36^. Increased expression of the CYP-4A family genes under the dose of HgCl _2_ and MeHg used in the present study could impact lipid metabolism.

CYP7A1 is a rate-limiting enzyme for bile acid synthesis and is regulated by FXR ^23^. Little is known on the effects of mercury compounds on FXR and CYP7A1 expression. The present study showed that 70W and Zuotai did not affect CYP7A1, while HgCl _2_ and MeHg increased Cyp7a1 mRNA and CYP7A1 protein. The biological effects of CYP7A1 induction by HgCl _2_ and MeHg warrant further investigation.

## Conclusions

The present study showed β-HgS and β-HgS containing 70W (1–5-times of clinical dose) did not produce appreciable effects on hepatic CYP450 enzyme gene/protein expression compared to equal Hg content as HgCl _2_ or 1/10 of Hg content as MeHg, suggesting that (1) the protection of 70W against CCl _4_ hepatotoxicity is not due to inhibition of CYP450 (CYP2E1); (2) 70W appeared to be safe under recommended clinical doses; and (3) HgCl _2_ and MeHg had significant effects on CYP450 expression, correlated with their potential toxic effects to the liver. 

## Abbreviations

70 Wei-Zhen-Zhu-Wan (70W, also called Rannasangpei; Qishiwei); Cytochrome P450 (CYP450); Aryl hydrocarbon receptor (AhR); Constitutive androstane receptor (CAR); Pregnane X receptor (PXR); Peroxisome proliferator-activated receptors (PPARs); farnesoid X receptor (FXR).

## Data availability

### Underlying data

Zenodo: Zuotai (β-HgS)-containing 70 Wei Zhen-Zhu-Wan differs from mercury chloride and methylmercury on hepatic cytochrome P450, http://doi.org/10.5281/zenodo.4403717 ^40^

This project contains the following underlying data:

- PCR and WB figure data (PCR-WB)
- Raw western-blot data (CYP-WB). Please note that full blot images are not available

Data are available under the terms of the Creative Commons Attribution 4.0 International license (CC-BY 4.0).

## References

[1] Schwabl H Vennos C : From medical tradition to traditional medicine: A Tibetan formula in the European framework. *J Ethnopharmacol.* 2015;167:108–114. 10.1016/j.jep.2014.10.033 25446636

[2] Huang HB Wang QZ Wang XW : [Overview of current researches on Tibetan medicine "zuotai"]. *Zhongguo Zhong Yao Za Zhi.* 2013;38(17):2886–2888. 24380317

[3] Kan ZB : [An introduction of Zuotai in Tibetan patent medicine]. *Zhongguo Zhong Yao Za Zhi.* 2013;38(10):1621–1623. 23947151

[4] Schwabl A Gämperle E : [Special aspects of quality of Tibetan medicines--insights from over 40 years of manufacturing experience in Switzerland]. *Forsch Komplementmed.* 2013;20 Suppl 2:14–16. 10.1159/000351071 23860107

[5] Reuter KP Weißhuhn TE Witt CM : Tibetan medicine: a systematic review of the clinical research available in the west. *Evid Based Complement Alternat Med.* 2013;2013:213407. 10.1155/2013/213407 23662117 PMC3638583

[6] Li Q Li H J Xu T : Natural Medicines Used in the Traditional Tibetan Medical System for the Treatment of Liver Diseases. *Front Pharmacol.* 2018;9:29. 10.3389/fphar.2018.00029 29441019 PMC5797630

[7] Liu J Zhang F Ravikanth V : Chemical Compositions of Metals in Bhasmas and Tibetan Zuotai Are a Major Determinant of Their Therapeutic Effects and Toxicity. *Evid Based Complement Alternat Med.* 2019;2019:1697804. 10.1155/2019/1697804 30941186 PMC6421027

[8] Hu AL Song S Li Y : Mercury sulfide-containing Hua-Feng-Dan and 70W ( *Rannasangpei*) protect against LPS plus MPTP-induced neurotoxicity and disturbance of gut microbiota in mice. *J Ethnopharmacol.* 2020;254:112674. 10.1016/j.jep.2020.112674 32105745

[9] Chinese Pharmacopoeia: Pharmacopeia of the People's Republic of China. *Chinese Medical Press.* 2015;1. Reference Source

[10] Wu P Luo Y Zhen L : Rannasangpei Is a Therapeutic Agent in the Treatment of Vascular Dementia. *Evid Based Complement Alternat Med.* 2016;2016:2530105. 10.1155/2016/2530105 27293454 PMC4886068

[11] Xu M Wu R Liang Y : Protective effect and mechanism of Qishiwei Zhenzhu pills on cerebral ischemia-reperfusion injury via blood-brain barrier and metabonomics. *Biomed Pharmacother.* 2020;131:110723. 10.1016/j.biopha.2020.110723 33152910

[12] Zhang BB Nie, Y Hu AL : Effect of Qishiwei pearl pills on intestinal microbiota in mice. *Zhong Cheng Yao.* 2020;42(3):626–631.

[13] Nie Y Zhang BB Du YZ : Protective effects of 70 Wei Zhen-Zhu-Wan against carbon tetrachloride induced liver injury in mice. *Zunyi Medical University.* 2018;40:358–363.

[14] Raucy JL Kraner JC Lasker JM : Bioactivation of halogenated hydrocarbons by cytochrome P4502E1. *Crit Rev Toxicol.* 1993;23(1):1–20. 10.3109/10408449309104072 8471158

[15] Jin SE Ha H Shin HK : Effects of traditional herbal formulae on human CYP450 isozymes. *Chin J Integr Med.* 2017;23(1):62–69. 10.1007/s11655-016-2476-3 27352178

[16] Parvez MK Rishi V : Herb-Drug Interactions and Hepatotoxicity. *Curr Drug Metab.* 2019;20(4):275–282. 10.2174/1389200220666190325141422 30914020

[17] Lu Y Xie T Zhang Y : Triptolide Induces hepatotoxicity via inhibition of CYP450s in Rat liver microsomes. *BMC Complement Altern Med.* 2017;17(1):15. 10.1186/s12906-016-1504-3 28056947 PMC5217299

[18] Xu S Liu J Shi J : 2,3,4',5-tetrahydroxystilbene-2-O- *β*-D-glucoside exacerbates acetaminophen-induced hepatotoxicity by inducing hepatic expression of CYP2E1, CYP3A4 and CYP1A2. *Sci Rep.* 2017;7(1):16511. 10.1038/s41598-017-16688-5 29184146 PMC5705655

[19] Aleksunes LM Klaassen CD : Coordinated regulation of hepatic phase I and II drug-metabolizing genes and transporters using AhR-, CAR-, PXR-, PPARα-, and Nrf2-null mice. *Drug Metab Dispos.* 2012;40(7):1366–1379. 10.1124/dmd.112.045112 22496397 PMC3382842

[20] Zhang BB Liu YM Hu AL : HgS and Zuotai differ from HgCl(2) and methyl mercury in intestinal Hg absorption, transporter expression and gut microbiome in mice. *Toxicol Appl Pharmacol.* 2019;379:114615. 10.1016/j.taap.2019.114615 31175882

[21] Wu Q Li WK Zhou ZP : The Tibetan medicine Zuotai differs from HgCl _2_ and MeHg in producing liver injury in mice. *Regul Toxicol Pharmacol.* 2016;78:1–7. 10.1016/j.yrtph.2016.03.017 27032305

[22] Zhang BB Li WK Hou WY : Zuotai and HgS differ from HgCl _2_ and methyl mercury in Hg accumulation and toxicity in weanling and aged rats. *Toxicol Appl Pharmacol.* 2017;331:76–84. 10.1016/j.taap.2017.05.021 28536007

[23] Liu J Lu H Lu YF : Potency of individual bile acids to regulate bile acid synthesis and transport genes in primary human hepatocyte cultures. *Toxicol Sci.* 2014;141(2):538–546. 10.1093/toxsci/kfu151 25055961 PMC4271050

[24] Liu J Wei LX Wang Q : A review of cinnabar (HgS) and/or realgar (As _4_S _4_)-containing traditional medicines. *J Ethnopharmacol.* 2018;210:340–350. 10.1016/j.jep.2017.08.037 28864167

[25] Li C Xu W Chu S : The chemical speciation, spatial distribution and toxicity of mercury from Tibetan medicine *Zuotai*, β-HgS and HgCl _2_) in mouse kidney. *J Trace Elem Med Biol.* 2018;45:104–113. 10.1016/j.jtemb.2017.08.010 29173465

[26] Li C Wang DP Duo J : [Study on safety of Tibetan medicine zuotai and preliminary study on clinical safety of its compound dangzuo]. *Zhongguo Zhong Yao Za Zhi.* 2014;39(13):2573–2582. 25276985

[27] Sallon S Dory Y Barghouthy Y : Is mercury in Tibetan Medicine toxic? Clinical, neurocognitive and biochemical results of an initial cross-sectional study. *Exp Biol Med (Maywood).* 2017;242(3):316–332. 10.1177/1535370216672748 27738246 PMC5384498

[28] Vickers AJ van Haselen R Heger M : Can homeopathically prepared mercury cause symptoms in healthy volunteers? A randomized, double-blind placebo-controlled trial. *J Altern Complement Med.* 2001;7(2):141–148. 10.1089/107555301750164208 11327520

[29] Yu YH Li LS : Effect of Rannasangpei on human liver and kidney function and blood biochemistry. *Shi-Zhen Guoyi Guoyao.* 2014;25:2848–2851.

[30] Liu J Lu YF Li WK : Mercury sulfides are much less nephrotoxic than mercury chloride and methylmercury in mice. *Toxicol Lett.* 2016;262:153–160. 10.1016/j.toxlet.2016.10.003 27720909

[31] Anwar-Mohamed A Elbekai RH El-Kadi AO : Regulation of CYP1A1 by heavy metals and consequences for drug metabolism. *Expert Opin Drug Metab Toxicol.* 2009;5(5):501–521. 10.1517/17425250902918302 19416086

[32] Xu SF Wu Q Zhang BB : Comparison of mercury sulfides with mercury chloride and methylmercury on hepatic P450, phase-2 and transporter gene expression in mice. *J Trace Elem Med Biol.* 2016;37:37–43. 10.1016/j.jtemb.2016.06.006 27473830

[33] Li XY Liu YN Li YP : [Effect of Tibetan medicine zuotai on the activity, protein and mRNA expression of CYP1A2 and NAT2]. *Yao Xue Xue Bao.* 2014;49(2):267–272. 24761621

[34] Zhen H Wen M Yang Y : Toxic effects of HgCl _2_ on activities of SOD, AchE and relative expression of *SOD*, *AChE*, *CYP1A1* of zebrafish. *Ecotoxicology.* 2014;23(10):1842–1845. 10.1007/s10646-014-1350-3 25240424

[35] Amara IEA Elshenawy OH Abdelrady M : Acute mercury toxicity modulates cytochrome P450, soluble epoxide hydrolase and their associated arachidonic acid metabolites in C57Bl/6 mouse heart. *Toxicol Lett.* 2014;226(1):53–62. 10.1016/j.toxlet.2014.01.025 24472606

[36] Lu YF Wu Q Liang SX : Evaluation of hepatotoxicity potential of cinnabar-containing *An-Gong-Niu-Huang Wan*, a patent traditional Chinese medicine. *Regul Toxicol Pharmacol.* 2011;60(2):206–211. 10.1016/j.yrtph.2011.03.007 21435368

[37] Hartman JH Miller GP Meyer JN : Toxicological Implications of Mitochondrial Localization of CYP2E1. *Toxicol Res (Camb).* 2017;6(3):273–289. 10.1039/C7TX00020K 28989700 PMC5627779

[38] Joshi D Mittal DK Shukla S : *Curcuma longa* Linn. extract and curcumin protect CYP 2E1 enzymatic activity against mercuric chloride-induced hepatotoxicity and oxidative stress: A protective approach. *Exp Toxicol Pathol.* 2017;69(6):373–382. 10.1016/j.etp.2017.02.009 28336172

[39] Amara IEA Anwar-Mohamed A Abdelhamid G : Mercury modulates the cytochrome P450 1a1, 1a2 and 1b1 in C57BL/6J mice: in vivo and in vitro studies. *Toxicol Appl Pharmacol.* 2013;266(3):419–429. 10.1016/j.etp.2017.02.009 23231920

[40] Nie Y Xu SF Lu YL : Zuotai (β-HgS)-containing 70 Wei Zhen-Zhu-Wan differs from mercury chloride and methylmercury on hepatic cytochrome P450 (Version 1).2020. 10.5281/zenodo.4403717 PMC8240600 34249337

