# Supplementary material for: Zuotai (β-HgS)-containing 70 Wei Zhen-Zhu-Wan differs from mercury chloride and methylmercury on hepatic cytochrome P450 in mice
Source: F1000Res. 2021 Jun 24;10:203. Originally published 2021 Mar 11. [Version 2] doi: 10.12688/f1000research.40667.2 (PMC8240600; doi:10.12688/f1000research.40667.2)
Supplement: Peer review history

# Peer review: Reviewer response for version 2

**DOI:** 10.5256/f1000research.58028.r88174

**Published:** 2021-06-28

**Authors:** Xingguo Cheng

**Affiliations:** 1 Department of Pharmaceutical Sciences, St. John's University, Queens, New York, NY, USA

The authors successfully addressed my comments. No more comments.

Is the work clearly and accurately presented and does it cite the current literature?

Yes

If applicable, is the statistical analysis and its interpretation appropriate?

Yes

Are all the source data underlying the results available to ensure full reproducibility?

Yes

Is the study design appropriate and is the work technically sound?

Yes

Are the conclusions drawn adequately supported by the results?

Yes

Are sufficient details of methods and analysis provided to allow replication by others?

Yes

Reviewer Expertise:

Toxicology

I confirm that I have read this submission and believe that I have an appropriate level of expertise to confirm that it is of an acceptable scientific standard.

# Peer review: Reviewer response for version 1

**DOI:** 10.5256/f1000research.43732.r81778

**Published:** 2021-04-30

**Authors:** Xian-Ju Huang

**Affiliations:** 1 College of Pharmaceutical Science, South-Central University for Nationalities, Wuhan, China

The article was well designed to discuss the hepatotoxicity of Zuotai-containing 70W and the data persuasive. However, some errors should be corrected.

1. The dose of 70W (0.15-1.5 g/kg) should be 1-10 times clinical doses. The author described as 1-5 times.
2. The data of ALT and AST as well as pathological changes were not shown in the results. However, the methods have mentioned. Please check them.
3. The introduction section, the second paragraph, line 2, "Pamda-28" may not be true, please check it.  

Is the work clearly and accurately presented and does it cite the current literature?

Yes

If applicable, is the statistical analysis and its interpretation appropriate?

Yes

Are all the source data underlying the results available to ensure full reproducibility?

Yes

Is the study design appropriate and is the work technically sound?

Yes

Are the conclusions drawn adequately supported by the results?

Yes

Are sufficient details of methods and analysis provided to allow replication by others?

Yes

Reviewer Expertise:

Pharmacology and toxicology

I confirm that I have read this submission and believe that I have an appropriate level of expertise to confirm that it is of an acceptable scientific standard.

# Peer review: Reviewer response for version 1

**DOI:** 10.5256/f1000research.43732.r81779

**Published:** 2021-03-30

**Authors:** Xingguo Cheng

**Affiliations:** 1 Department of Pharmaceutical Sciences, St. John's University, Queens, New York, NY, USA

Because of their toxic components and potential drug-drug interaction, many traditional Asian Medicine, including Tibetan Medicine, have been raising health concerns. In this manuscript, Nie et al evaluated and compared the expression regulation of several P450s by Zuotai, mercury chloride and methylmercury, given the same mercury content level. The authors suggested that it is not appropriate to simply use total Hg content to evaluate the safety of HgS-containing Zuotai. Overall, the study is straight-forward. 

  

1. Figure legend of Figure 1: should be total RNA from mouse liver was extracted and processed for RT-PCR analysis.
2. In animal treatment section, the mice should be treated with Zuotai, mercury chloride and methylmercury by using the unit of mmole/kg or micromole/kg, but not mg/kg unit.
3. The authors have assessed Cyp7a1 and FXR expression. To make the studies more relevant, the authors may consider to measure total bile acids in mouse serum, a biomarker of liver injury.

Is the work clearly and accurately presented and does it cite the current literature?

Yes

If applicable, is the statistical analysis and its interpretation appropriate?

Yes

Are all the source data underlying the results available to ensure full reproducibility?

Yes

Is the study design appropriate and is the work technically sound?

Yes

Are the conclusions drawn adequately supported by the results?

Yes

Are sufficient details of methods and analysis provided to allow replication by others?

Yes

Reviewer Expertise:

Toxicology

I confirm that I have read this submission and believe that I have an appropriate level of expertise to confirm that it is of an acceptable scientific standard, however I have significant reservations, as outlined above.

# Response

**Published:** 2021-04-30

**Authors:** Jie Liu

**Affiliations:** Zunyi Medical College, USA

Thanks for the comments.  Some of the inquiries could be found in listed references Nie et al., 2018.

# Response

**Published:** 2021-04-30

**Authors:** Jie Liu

**Affiliations:** Zunyi Medical College, USA

1. Figure legend of Figure 1: should be total RNA from mouse liver was extracted and processed for RT-PCR analysis.
   Answer: Yes
2. In animal treatment section, the mice should be treated with Zuotai, mercury chloride and methylmercury by using the unit of mmole/kg or micromole/kg, but not mg/kg unit.  Answer: Since Zoutai is a mineral mixture, we can only use mg/kg.  mercury chloride and MeHg were used based on mol basis as HgS
3. The authors have assessed Cyp7a1 and FXR expression. To make the studies more relevant, the authors may consider to measure total bile acids in mouse serum, a biomarker of liver injury. Answer: Good suggestion for future consideration.  ALT and histology in prior publications verified the toxicity
